# Supplementary material for: The magnitude and associated factors of immediate postpartum anemia among women who gave birth in east Gojjam zone hospitals, northwest- Ethiopia, 2020
Source: PLoS One. 2023 Mar 15;18(3):e0282819. doi: 10.1371/journal.pone.0282819 (PMC10016639; doi:10.1371/journal.pone.0282819)
Supplement: S1 File — (ZIP) [file pone.0282819.s002.zip › approval mar5/DocScanner Mar 3, 2023 1-44 PM.pdf]

በኢትዮጵያ ፌደራላዊ ዲሞክራሲያዊ ሪፐብሊክ
የትምህርት ሚኒስቴር
ደብረ ማርቆስ ዩኒቨርሲቲ
ምር/ማህ/አገ/እና ድህረ ምረቃ አስተባባሪ

The Federal Democratic Republic Of Ethiopia
Ministry Of Education
Debre Markos University
Res/Com/ser/&Post graduated coordinating office

ቁጥር፡ጤሳኮ/ም/ማ/አገ/ድ/ማስ/34/11/13

ቀን፡29/01/2013 ዓ.ም

ለሉማሜ ሆስፒታል
ለሸበል በረንታ ሆስፒታል
ለቢቸና ሆስፒታል
ለመርጦለማርያም ሆስፒታል
ሞጣ ሆስፒታል

ጉዳዩ፡- **ትብብር ይመለከታል፣**

በደብረ ማርቆስ ዩኒቨርሲቲ በጤና ሳይንስ ኮሌጅ የሁለተኛ ዲግሪ ተማሪ የሆነው/ችው/ ጌታቸው አልታሰብ በዩንቨርሲቲው አቅርቦ/ባ/ የፀደቀለት/ላት/ የምርምር ስራ " Magnitude of immediate post partum anemia and associated factors among women who give birth in East Gojjame Zone Hospitals North Weast Ethiopia 2020" በሚል ርእስ በማካሄድ ላይ በመሆኑ/ኗ/ መረጃ ለማሰባሰብ ከጥናቱ ጋር በተያያዘ የጥናቱ የመረጃ ሰብሳቢ ባለሙያ አባላት ሲንቀሳቀሱ በእናንተ በኩል አስፈላጊውን ትብብር እንዲደረግላቸው እያልን ስለሚደረግላቸው ትብብር ከወዲሁ እናመሰግናል፡፡

"መማር መመራመራችን ለማህበረሰባችን!"

በቃሉ ካሴ ዓለሙ
Bekalu Kassie Alemu

የምርምር ማህበረሰብ አገልግሎትና
ድህረ-ምረቃ አስተባባሪ
Research Community Service
& Postgraduate Coordinator

ግልባጭ፣

- ለአቶ ጌታቸው አልታሰብ

891

☎ 058-778-0844
Fax: 058-771-1764
E-mail dmuhsc@yahoo.com
✉ 269
መልስ ሲጽፉልን እባክዎን የኛን ቁጥር ይጥቀሱ
In replying Please Quote our Ref.No.
