# Supplementary material for: The magnitude and associated factors of immediate postpartum anemia among women who gave birth in east Gojjam zone hospitals, northwest- Ethiopia, 2020
Source: PLoS One. 2023 Mar 15;18(3):e0282819. doi: 10.1371/journal.pone.0282819 (PMC10016639; doi:10.1371/journal.pone.0282819)
Supplement: S1 File — (ZIP) [file pone.0282819.s002.zip › approval mar5/ethical approval PDF.pdf]

The Federal Democratic Republic of Ethiopia Ministry of Education
Debre Markos University
Res/ComSer/& post graduated coordinating office

To:Lumame primary Hospital

Shebel Berenta primary Hospital

Bichena primary Hospital

Mertolemariam primary Hospital

Motta primary Hospital

Ref no : HSC/R/C/Scr/Co/34/11/13

Date: 29/01/2013 E.C

Subject: **Ethical Approval of research study**

The institutional review committee in its meeting held on 29th September, 2013 Ethiopian calendar has reviewed and discussed the application submitted to conduct the research proposal entitled**" Magnitude of immediate postpartum anemia and associated factors among women who gave birth in East Gojjam Zone Hospitals ,Northwest Ethiopia 2020: institutional based cross-sectional study"**

EC Decision: Provisional Approved

Kindly quote the above reference code in all further communications regarding the above subject.

CC

-Mr. Getachew Altaseb

"Education and research for community service"

Bekalu Kassie Alemu

Research Community service &Postgraduate Coordinator

Phone no 05-778-0844
Fax 058771-1764
Po.Box 269
E-mail dmuhsc@yahoo.com
