# Supplementary material for: The magnitude and associated factors of immediate postpartum anemia among women who gave birth in east Gojjam zone hospitals, northwest- Ethiopia, 2020
Source: PLoS One. 2023 Mar 15;18(3):e0282819. doi: 10.1371/journal.pone.0282819 (PMC10016639; doi:10.1371/journal.pone.0282819)
Supplement: Attachment — Submitted filename: Comments to authors.docx [file pone.0282819.s003.docx]

Reviewer comments:

Title:  Magnitude and associated factors of immediate postpartum anemia among women who gave birth in east Gojjam zone hospitals, northwest- Ethiopia, 2020

The manuscript raised important public health problems in low and middle income countries, especially in Ethiopia, but the documents need thorough work on to reshape and make clearer to readers.

1.The study presents the results of original research.

Yes

2. Results reported have not been published elsewhere.

Yes

3. Experiments, statistics, and other analyses are performed to a high technical standard and are described in sufficient detail.

Yes

4. Conclusions are presented in an appropriate fashion and are supported by the data.

Yes

5. The article is presented in an intelligible fashion and is written in standard English.

No: the document need thorough English language edition.

6. The research meets all applicable standards for the ethics of experimentation and research integrity.

Yes

7. The article adheres to appropriate reporting guidelines and community standards for data availability.

Yes

Specific comments

Abstract

Background**:** line 20, “less or equal to 10 g/dl in”, but according to World Health Organization (WHO), anemia is defined as hemoglobin (Hb) levels <12.0 g/dL in women. Do you have explanation for the different definition?

Conclusion: line 41, how much is “a moderate public health problem”? 21.6? What is the implication of your finding?

Introduction: why do you classified introduction in two background and statement of the problems? Further, the so called background was not well articulated.

Line 76, “anemia in the developed country ranges from 10-30% and in developing country 50-80%”, it is preferable if data from low and middle income countries used instead of developing countries. What is the global burden? The citation are too old to put current image of the problems (for instance, reference 10, 11, 12). Please update other references accordingly.

## The Excerpt you cited have no the idea you mentioned in the documents i.e., “line 84-89” or reference 16 or “<https://pubmed.ncbi.nlm.nih.gov/27227230/>”

Line 92-95, are those consequence of anemia serious enough to design this study?

Line 114, “assessing the burden of disease” how health facility-based study reveal burden?

What did your study add to existing knowledge? In addition to what Abebaw et al, 2020 were explored.

Sample size and sampling procedure: was sample size calculated for factors? If no why?

Why multistage? How many stages were there? Why design effect of 1.5?

Line 139, “every 2 case intervals” how did you find 2? Why not 3?

Data collection procedures and instrumentations: cite at least few references from where you adopted the questionnaire.

The variables measurement need major revision. Laboratory procedure also need further explanation. How blood sample was taken? Who take the blood sample? How blood sample was transported? Etc.

Result: line 184-185, age explained in three ways. Avoid redundancy.

Bivariable and multivariable logistic regression analysis: where are explanation of multivariable analysis?

Discussion: the discussion begin with summary findings. If your study is in line with “Amhara region, Northwest Ethiopia 24.3% (23), Tigray region Northwest Ethiopia 24.2% (24), Germany 25%(25), North Carolina, USA 19.1%(9)” what it add to existing knowledge?

“different scholars used different cutoff values for hemoglobin measurements to determine postpartum anemia.” Why you compare with already different study? But you didn’t mention “Another possible reason may be mothers who develop anemia during their pregnancy were not included in this study.” in your exclusion?

Your discussion should look different from result.

The discussion need through writing, highlighting the implication for policy.

The references style was not plos one style and need correction and updating.

What are the strength and limitation of your study?

Conclusion: What are the policy implication of the documents?
