# Supplementary material for: The magnitude and associated factors of immediate postpartum anemia among women who gave birth in east Gojjam zone hospitals, northwest- Ethiopia, 2020
Source: PLoS One. 2023 Mar 15;18(3):e0282819. doi: 10.1371/journal.pone.0282819 (PMC10016639; doi:10.1371/journal.pone.0282819)
Supplement: Attachment — Submitted filename: Response to Reviewers.docx [file pone.0282819.s004.docx]

Response to Reviewers

Response to Academic Editor

1. When submitting your revision, we need you to address these additional requirements. Please ensure that your manuscript meets PLOS ONE’s style requirements, including those for file naming.

**Response**: we carefully reviewed the PLOS ONE’s requirement, and manuscript is stick to the outline given under Author’s guideline.

2. We suggest you thoroughly copyedit your manuscript for language usage, spelling and grammar.

**Response**: We reviewed the entire document thoroughly for the English Language usage, spelling, grammar and the necessary changes were made.

3. Thank you for stating the following in the acknowledgement/funding section for your manuscript, please note that the funding the information should not appear in the acknowledgement section

**Response:** sorry that we have mentioned funding information on the acknowledgment section in our original submission and now we removed funding related texts in the revised manuscript.

4.  We note that you have stated that you will provide repository information for your data at acceptance. Should your manuscript be accepted for publication, we will hold it until you provide the relevant accession numbers or DOIs necessary to access your data. If you wish to make changes to your Data Availability statement, please describe these changes in your cover letter and we will update your Data Availability statement to reflect the information you provide

**Response:** sorry it was wrongly ticked while selecting during the online submission, otherwise all data are fully available without restriction.

5.   Thank you for submitting the above manuscript to PLOS ONE. During our internal evaluation of the manuscript, we found significant text overlap between your submission and the following previously published works, some of which you are an author.

<http://repository-tnmgrmu.ac.in/8960/1/201500418samykkhan.pdf>
<https://www.omicsonline.org/open-access/postpartum-anemia--still-a-major-problem-on-a-global-scale-2376-127X-1000e122.php?aid=61457>
<https://www.hindawi.com/journals/anemia/2020/8979740/>

We would like to make you aware that copying extracts from previous publications, especially outside the methods section, word-for-word is unacceptable. In addition, the reproduction of text from published reports has implications for the copyright that may apply to the publications.

Please revise the manuscript to rephrase the duplicated text, cite your sources, and provide details as to how the current manuscript advances on previous work. Please note that further consideration is dependent on the submission of a manuscript that addresses these concerns about the overlap in text with published work.

Response: we revised the manuscript and update it by avoiding duplicity of the ideas and tried to bear with original work with intended purposes to be under each section.

Response Reviewers’ comments:

1. Is the manuscript technically sounds, and do the data support the conclusions?

Response: the manuscript is technically sounds and the data drawn based on the data presented

2. Has the statistical analysis been performed appropriately and rigorously?

Response: statistical analysis been performed with appropriate model of regression and rigorously

3. Have the authors made all data underlying the findings in their manuscript fully available?

Response: all data are available fully in the manuscript

4. Is the manuscript presented in an intelligible fashion and written in Standard English?

Response: We reviewed the entire document thoroughly for the English Language usage, spelling, grammar in an intelligible fashion and the necessary changes were made based on the comment given

Background: line 20, “less or equal to 10 g/dl in”, but according to World Health Organization (WHO), anemia is defined as hemoglobin (Hb) levels <12.0 g/dL in women. Do you have explanation for the different definition?

Response: according to WHO definition of Anemia in a women is Hgb levels <12.0g/dl, but during pregnancy there is physiologic change that means there is an increase both in red blood cell and plasma level despite of this the increase in red blood cell by about 33% and plasma level by about 50% is not proportional that leads to hem dilution and drop of the Hgb level during pregnancy, so that is the reason the cutoff point Hgb <=10g/dl to define anemia and it doesn’t regain its non-pregnant state Hgb level

 How much is “a moderate public health problem”? 21.6? What is the implication of your finding?

Response: based on its public health importance postpartum anemia can be classified as normal mild, moderate and severe according to its prevalence. If the prevalence is ≤4.9% it is normal no public health problem, 5-19.9% is mild public health problem, 20-39.9% is moderate public health problem, ≥40% severe public health problem, so the prevalence of the is finding is 21.6% and put it under moderate public health problem as per to the classification needs attention to avert because of it public health problem.

Why do you classified introduction in two background and statement of the problems? Further, the so called background was not well articulated.

Response: we will revise and make the two sections of the introduction

What did your study add to existing knowledge? In addition to what Abebaw et al, 2020 were explored

Response: environmental factors were not assessed from previous studies and for immediate post-partum anemia Hgb measurement is taken from cutoff point 10-10.5g/dl in WHO recommendation but in the previous studies used the cut point of 11g/dl at one week of postpartum other than immediate postpartum that affects the overall image of the finding.

Sample size and sampling procedure: was sample size calculated for factors? If no why?

Response; sample size for the factor was calculated by using Epi-Info statclc

Why multistage? How many stages were there? Why design effect of 1.5?

Response: there are ten Hospitals in the study area of them five were selected randomly by using lottery method then study participants were taken from five hospital which means we go dawn from ten to five the proportional allocation was used and has two stage, based on the sage we can use design effect of two but due to its cost wise we used design effect 1.5

“every 2 case intervals” how did you find 2? Why not 3?

Response: K- interval, K= $\frac{N}{n}$ , where: N = Total population immediate post-partum at the selected five Hospitals , n=Total sample size, then k=1,065/467 = approximately every 2 mothers will take but, calculating K-value for each hospital was necessary by using K1=$\frac{N1}{n1}$, K2=$\frac{N2}{n2}$……….(K= 2.27, 2.24 ,2.25 …. approximately every 2 immediate postpartum women was taking for each hospital after the first case selecting randomly between 1 and K). if we take 3 it becomes 1401 which is more than the Total population immediate post-partum at the selected five Hospitals due to this we took 2

If your study is in line with “Amhara region, Northwest Ethiopia 24.3% (23), Tigray region Northwest Ethiopia 24.2% (24), Germany 25%(25), North Carolina, USA 19.1%(9)” what it add to existing knowledge

Response: despite the fact that the magnitude of the study is in line with these previous studies environmental factors were not assessed from previous studies.

“different scholars used different cutoff values for hemoglobin measurements to determine postpartum anemia.” Why you compare with already different study? But you didn’t mention “Another possible reason may be mothers who develop anemia during their pregnancy were not included in this study.” in your exclusion?

Response: in our study mothers who were anemic in their ANC follow up were assessed and excluded but previous studies were not exclude mothers who were anemic during their ANC follow up that is we want to explain but not compare studies done on pregnant, mothers

Your discussion should look different from result

Response: we revised the discussion session

Reviewer #2:

When the data collected (blood specimen)? No time limit and question inclusion and exclusion criteria was not mentioned Is there a difference in cut of point to define anemia after delivery? Based on the duration? If so how managed?

Response: the data were collected with 24 hours of post- delivery, yes the time matters after delivery to define post-partum anemia , based on WHO current recommendation for immediate post-partum /within 24 hours of anemia the cutoff point is 10g/dl whereas as at one week of post-partum 11g/dl at eight weeks of post-partum 12 g/dl is used to determine anemia after delivery and the management is based on the time and level of Hgb

How data collection of dietary and micronutrient utilization conducted? How co-existing infection collected and measured?

Response: by using adapted questioners like Irion and folic acid intake during pregnancy, for how many times took iron and folic acid supplementation in this Pregnancy, Have you drink coffee and tea when taking IFA Average Frequency of meal per day and MUAC measurement Helminths infestation, known past medical disease
