# Supplementary material for: The magnitude and associated factors of immediate postpartum anemia among women who gave birth in east Gojjam zone hospitals, northwest- Ethiopia, 2020
Source: PLoS One. 2023 Mar 15;18(3):e0282819. doi: 10.1371/journal.pone.0282819 (PMC10016639; doi:10.1371/journal.pone.0282819)
Supplement: Attachment — Submitted filename: Response to Reviewers R2.edited.docx [file pone.0282819.s006.docx]

**Response to Reviewers’ comments**

Reviewer #1:

1. The papers seem improved, but there are a lot of things that authors should consider to improve the manuscript before publishing it. The paper need thorough English language edition. Grammar, conjunctions and punctuation need correction. The gap of the study should be clearly stated in relation to what others failed to achieve.

Response: We reviewed the entire document thoroughly for the English Language usage, spelling, grammar, and the necessary changes were made.

-the sample size is adequate

2. What other studies identified are explained insufficiently.

The authors failed to show the gap in the study. Although they claimed environmental factors were not assessed in the author's response. What makes this study different from Abebaw et al., 2020 study?

Response: We included the gap between other previous studies, in the revised manuscript of this study.

3. Variables and measurements are absent. There are too many variables. Do you think your sample size is adequate? How quality of the laboratory test was maintained? Is an automated blood analyzer cell dyne 1800 appropriate test? How about its sensitivity and specificity?

Response: sorry that we have mentioned automated blood analyzer cell dyne 1800 wrongly we used the mindray hematology analyzer in the revised manuscript.

- We reviewed the entire document thoroughly for the English Language usage, spelling, grammar in an intelligible fashion and the necessary changes were made based on the comment given

- We minimized the use of abbreviations in the table and, we added the list of

Acronyms we used throughout the manuscript.

Reviewer #2:

The question I have raised and responded by the Authors need to be incorporated in the manuscript

1. A difference in cut of point to define anemia after delivery? Based on the duration? If so how managed? Need to be incorporated in methodology part how practically data collected or measured

Response: We included the cut off point to define anemia after delivery in the revised manuscript

2. How data collection of dietary and micro nutrient utilization conducted? How co-existing infection collected and measured?

Response: we included the way how data were collected in the revised manuscript
