# Supplementary material for: The magnitude and associated factors of immediate postpartum anemia among women who gave birth in east Gojjam zone hospitals, northwest- Ethiopia, 2020
Source: PLoS One. 2023 Mar 15;18(3):e0282819. doi: 10.1371/journal.pone.0282819 (PMC10016639; doi:10.1371/journal.pone.0282819)
Supplement: Attachment — Submitted filename: Response to Reviewers R3.edited.docx [file pone.0282819.s007.docx]

**Response to Reviewers’ comments**

1. My general comment is there is much evidence in the country on the area. The evidence generated were already explored by previously conducted researchers in different part of the countries example Amhara region similar region and Tigray region the study lacks novelty and gap not well addressed.

Response: We included the gap between other previous studies, in the revised manuscript of this study and there is a difference in topography in this study area and previous studies despite being in the same region, and anemia is affected by geographic location.

2. Introduction not focus on the study objectives PP anemia, the characteristics of the study area were not addressed. There are many related study authors have mentioned in Ethiopia, why study needed since Ethiopia context is similar?

Response: We included the gap between other previous studies, in the revised manuscript of this study, and the study area is characterized in the revised manuscript.

3. Sampling technique is not clear there was missed information, check it how systematic random sampling method Data were used since PNC in Ethiopia is very low? When blood collected and hemoglobin measured not clarified, how anemia identified depending on the duration after delivery the standard not clarified Co-existing disease-related variables mentioned (malaria, HIV/AIDS, tuberculosis) why important hook work infection not addressed since the authors mentioned bare foot?

Response: sampling technique is stated in the revised manuscript; the data were collected before mothers had been discharged to home and collected eight hours after delivery, we considered that hook warm infestations are explained by the bare boot

4. What quality assurance measure taken for blood collection and determining hemoglobin level?

Response: one-day training was given to data collectors and supervisors before they went to collect the data about ways of data collection and how to transfer to the laboratory

5. What measure taken for identified anemic women on ethical issue

Response: After determination of hemoglobin therapeutic dose of iron was supplemented before discharge for all anemic women

6. Result part marital status n=465 why? Episiotomy, perinatal tear check all result

Table quality need modification

Response: sorry it is editorial problem we checked and corrected down to the document, episiotomy and perineal tear were taken among vaginal deliveries only not cesarean deliveries

7. Among these mothers who took IFA, 68.3% had poor adherence can we say poor adherence by collecting retrospective data , what is the standard to measure adherence what data collection method authors have to use?

Response: supplementation was extracted from their chart and adherence was taken from an interview of the mothers

8. Language edition need further work

Response: We reviewed the entire document thoroughly for the English Language usage, spelling, and grammar in an intelligible fashion, and the necessary changes were made based on the comment given
