# Supplementary material for: The magnitude and associated factors of immediate postpartum anemia among women who gave birth in east Gojjam zone hospitals, northwest- Ethiopia, 2020
Source: PLoS One. 2023 Mar 15;18(3):e0282819. doi: 10.1371/journal.pone.0282819 (PMC10016639; doi:10.1371/journal.pone.0282819)
Supplement: Attachment — Submitted filename: Reviewer comments.docx [file pone.0282819.s005.docx]

Reviewer comments

The papers seems improved, but there are a lot things that authors should consider to improve the manuscript before publishing it. The paper need thorough English language edition. Grammar, conjunctions and punctuation need correction. The gap of the study should be clearly stated in relation to what others failed to achieve.

Abstract

Line 35: delete the reference. Begin the sentence appropriately.

Line 35-39: Be consistent and make every numbers two decimal point.

Introduction

What other studies identified are explained insufficiently.

The authors failed to show the gap of the study. Although they claimed environmental factors were not assessed in the author response. What makes this study different from Abebaw et al., 2020 study?

Method section

Variables and measurement are absent. There are too many variables. Do you think your sample size is adequate?

How quality of laboratory test was maintained? Is automated blood analyzer cell dyne 1800 appropriate test? How about its sensitivity and specificity?

Result

Line 184: Table number is missing.

Line 199-202: How did you verified the women have toilet facility? What do you mean by wearing shoes always in their daily activities? I didn’t see any operational definition in this document.

Line 204-207: what is the difference between two sentences?

Line 208: rewrite the title.

In the tables some cell of the tables is very few, you better consider re-categorizing them. The table legend should include abbreviations. For instance SVD? IAVD?

Line 213-219: table 4 cited twice.

Discussion

Is it fair to compare Ethiopia to German, USA? You better compare with LMICs.

The discussion seems result section, please interpret and implicate the finding. You should avoid odds ratio in the discussion section.

Limitation: What other potential limitation do you encountered? What about recall and measurement bias?
